# Green synthesis of CuO nanoparticles from *Punica granatum* leaves and their application in the enhancement of cold-pressed calcareous alkali-activated materials for sustainability

**DOI:** 10.1371/journal.pone.0336812

**Published:** 2025-11-21

**Authors:** Meera Salama, Pwadubashiyi Coston Pwavodi

**Affiliations:** 1 Department of Bioengineering, Faculty of Engineering, Cyprus International University, Haspolat, Nicosia, Mersin 10, Türkiye; 2 Department of Biomedical Engineering, Faculty of Engineering, Cyprus International University, Haspolat, Nicosia, Mersin 10, Türkiye; Universiti Tun Hussein Onn Malaysia, MALAYSIA

## Abstract

The green synthesis of metal nanoparticles using plant extracts offers an environmentally friendly approach that reduces energy consumption and avoids hazardous chemicals, thus increasing various sustainable applications. This study investigates the green synthesis of copper oxide nanoparticles (CuO nanoparticles) using extracts from pomegranate (*Punica granatum*) leaves and their application in enhancing calcareous alkali-activated materials (AAMs). Characterization techniques, including Ultraviolet-Visible spectroscopy (UV-Vis), Fourier-Transform Infrared Spectroscopy (FTIR), X-ray Diffraction (XRD), Dynamic Light scattering (DLS), and Transmission Electron Microscopy (TEM), were employed. CuO nanoparticles were incorporated into cold-pressed AAMs at varying concentrations (0.25%, 0.5%, and 1% by weight). Mechanical and physical properties such as compressive strength, porosity, and water absorption were evaluated, followed by microstructural analysis using XRD, FTIR, Scanning Electron Microscopy (SEM), and Energy-Dispersive X-ray Spectroscopy (EDX). The synthesized CuO nanoparticles exhibited high crystallinity with minor amorphous content and a narrow size distribution, with an average particle size of 78 nm. The incorporation of 0.5% CuO nanoparticles yielded optimal results, increasing compressive strength (UCS) by 37.3% and reducing porosity and water absorption by 0.75% and 0.4%, respectively, compared to the control. Microstructural analysis indicated that CuO nanoparticles enhance matrix compactness and promote the formation of alkali-activated binder phases. Overall, the findings demonstrate that *Punica granatum* leaf extract can be effectively used for the green synthesis of semi-spherical CuO nanoparticles (<100 nm) with good stability. Their incorporation into calcareous AAMs significantly improves matrix densification and mechanical performance.

## Introduction

Metallic nanoparticles can be manufactured utilizing several physical and chemical approaches, such as sonication, thermal breakdown, and chemical or electrochemical reduction procedures [1,2]. However, these conventional technologies frequently suffer from restrictions such as excessive energy consumption and harmful chemicals, which pose risks to human health and the environment [2,3]. To address these challenges, chemical reduction procedures are increasingly classified as conventional and green synthesis methods [1]. Traditional chemical processes frequently involve hazardous chemicals, often harmful to the environment and human health. Green chemical synthesis procedures are recommended for simplicity, cost-effectiveness, and environmental friendliness [3–6].

Under mild reaction conditions, green chemistry allows for the synthesis of CuO nanoparticles of uniform sizes and different shapes. Plant extracts from leaves, roots, stems, bark, flowers, fruits, and seeds, among others, are utilized as reducing and/or stabilizing agents [5,7–13]. Plant extracts, which are cost-effective and easy to use, are particularly noteworthy among these options. Plant extracts are rich in antioxidant substances such as flavonoids, amino acids, and organic acids, which act as stabilizing agents in the synthesis of metal nanoparticles [14]. One such plant extract is from *Punica granatum*, known as pomegranate, which is known for its high concentration of antioxidant compounds and successful use in green synthesis.

Leaf extracts are particularly noteworthy among other non-edible plant components since they have a larger phytochemical concentration than other sections, such as the stem, root, or peel [15]. For instance, Rahayu and Lestari [16] reported that the total phenolic content of the leaf extract was substantially higher than that of the stem extract. Jaafar Karimi [17] found that the leaf extract of *Punica granatum* has the greatest total phenolic and flavonoid contents compared to the root and stem extracts. Also, Akkawi, Abu-lafi [18] revealed that the leaf extract had better bioactivity than other plant parts.

Furthermore, comparative studies suggest that the leaf section of *Punica granatum* is an extremely rich source of phytochemicals [15,19]. Yu, Gouvinhas [15], evaluated the leaf extracts of numerous plants, including peppermint, sage, rosemary, and parsley, and found that *Punica granatum* had the greatest total phenolic concentration. These findings highlight the potential of *Punica granatum* leaf extracts in green synthesis, especially given their high antioxidant content. Green-synthesized nanoparticles derived from *Punica granatum* extracts have potential applications in the construction industry. Nanomaterials can be employed specifically to improve the qualities of alkali-activated building materials [20]. The *P. granatum* (pomegranate) leaves are unusually rich in polyphenols, flavonoids, and other antioxidants, which makes them be used as effective reducing and capping agents for the conversion of metal ions to metal oxide nanoparticles compared with many other plant parts (peel, stem, root) and many other species. Taking advantage of the leaves also improves sustainability [8].

AAMs are gaining appeal as alternatives to standard cement-based materials due to their favorable features and low environmental effect. Alkali activation emerged in 1908 with the activation of slag, and then garnered substantial interest in laboratory research during the 1940s [21]. Alkali activation and its subgroup, geopolymers, are subjects of considerable research, focusing on their dissolution processes, gel formation, and hardening mechanisms [22–24]. For effective alkali activation, the precursor material must be rich in alumina and silica, with calcium playing a vital role. Successful alkali activation has been achieved using fly ash, metakaolin, slag, and rice husk ash [25]. Alkali activation typically involves silicate and hydroxide activators. The characteristics of AAMs are influenced by several parameters, some but not limited to the liquid-to-solid ratio, the molarity of the hydroxide activator, and the silicate-to-hydroxide ratio of the activators [21,22,25,26]. However, significant challenges remain: fluctuation in precursor availability across locations, the corrosiveness of hydroxide activators, and the high cost associated with using high molar ratios needed for dissolution [27].

The cold press method for compaction addresses some of these challenges by enhancing material structure while reducing the activator-to-solid ratio, lowering the amount of activator needed. Several researchers have investigated the impact of molding pressures on the mechanical properties of alkali-activated brick [28–32]. On the other hand, there have been several studies investigating the use of calcium carbonate-based materials as precursors for the alkali activation process, such as limestone [33–38], marble dust [39,40], seashell [41], and calcium carbonate [42,43]. These studies aim to utilize abundant calcareous materials, which comprise roughly 15% of the Earth’s crust. Despite their crystallinity, calcareous materials have shown improvements in strength when subjected to alkali activation.

Nanomaterials have been effectively used to improve the mechanical properties of alkali-activated materials. Specifically, nanosilica, nanoalumina, nanotitanium, and nanoclay have all been used to enhance these materials, accounting for 46%, 7%, 8.4%, and 11.4% of studies, respectively [20]. However, the use of CuO nanoparticles in AAMs remains limited. In a study conducted by Vavouraki, Gounaki [44], the inclusion of 0.5% CuO nanoparticles led to a 75% increase in the compressive strength of alkali-activated concrete and resulted in a denser structure. The observed enhancement is suggested to be due to the acceleration of alkali activation and the encouragement of denser gel formation, rather than the pozzolanic activity of the nanoparticles alone. The improved cohesion in the alkali-activated microstructure, which enhances the aggregate-paste bond, is largely attributed to the filler action of the nanoparticles [20]. Green-synthesized CuO nanoparticles with calcareous AAMs are combined through cold-pressing for material performance: CuO in nanoscale sizes acts as a high-surface-area filler and nucleation site that accelerates and increases binder gel formation that causing improved microstructure, reduced porosity, and higher compressive strength [44]. Sustainability that involves the use of calcareous wastes such as limestone, marble dust, and seashells forming AAMs from such Ca-rich precursors [33]. Process and economy using the cold-pressing method, which reduces required activator liquid, lowers efflorescence and alkali consumption, and produces denser products at room temperature or low curing energy [30].

This study is the first, to our knowledge, and based on the literature already published, to use *green-synthesized* CuO nanoparticles produced from *Punica granatum* leaf extract and then incorporate the synthesized CuO nanoparticles into calcareous alkali-activated materials that are consolidated by cold-pressing. This combination is not reported in other recent literature, and it addresses two research gaps simultaneously, which are sourcing sustainable nanoparticles and low-alkali and pressure-assisted production of AAMs based on abundant calcareous materials.

The research gaps filled by the current study are the following: Most of the studies done on nanoparticle-AAM employed the use of silica or alumina-rich precursors, such as fly ash, metakaolin, and slag; meanwhile, calcareous carbonate-rich precursors are less explored and react differently under alkali activation [33,45]. The addition of CuO nanoparticles to inorganic alkali systems is limited, and there are no reports that clearly state the combination of green-synthesized CuO with cold-pressed calcareous AAMs [44]. Lastly, the cold-pressing method for geopolymers and AAMs is a recent approach that is used to reduce the requirement of activator and to improve density. But specifically, studies that combine cold-pressing with nanoparticle reinforcement, such as CuO in calcareous matrices, have not yet been published [30].

The current study aims to enhance the properties of calcareous AAMs by incorporating green-synthesized CuO nanoparticles derived from *Punica granatum* leaves and employing pressure molding techniques. After a careful literature review, it has been discovered that there have been no prior attempts to improve calcareous AAMs using nanoparticles and/or molding pressure. This research seeks to address the limitations of previous approaches while enhancing the mechanical properties and environmental sustainability of calcareous alkali-activated materials.

## Materials and methods

### Collection and authentication of plant material

Fresh pomegranate leaves were collected from a nearby tree in Hamitköy, Lefkoşa, Nicosia, North Cyprus (35° 12’ 43“ N, 33° 22’ 36” E) and identified by the Pharmacognosist, Asst. Prof. Dr. Emmanuel Mshelia Halilu, at the Pharmacy Faculty, Cyprus International University. A sample voucher designated CIU/PHARM/PUNI/001 was deposited in the Cyprus International University’s herbarium for future reference.

### Ethics and consent approval

Local ethical permissions were obtained from the local authorities before collecting the plant used for the study.

### *Punica granatum* leaves extract preparation

**Fig 1:**
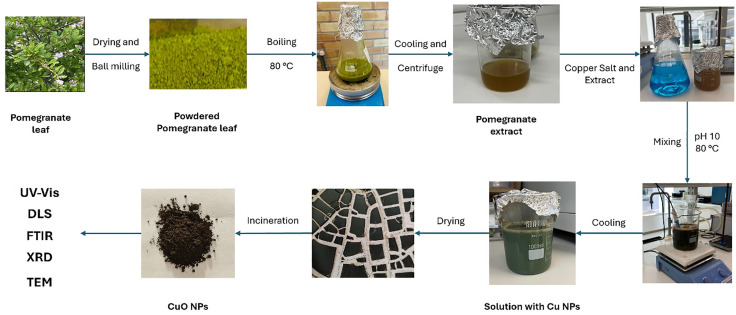
*Punica granatum* leaves and CuO nanoparticles synthesis procedure.

The leaves were carefully cleansed with deionized water before drying in an oven at 40 °C for three days. After drying, they were ground into a fine powder with a ball mill. To make the pomegranate leaf aqueous extract, 100 g of fine powder was soaked in deionized water and agitated with a magnetic stirrer at 80 °C for 15 minutes. The mixture was then allowed to cool to room temperature before centrifugation, which formed a yellowish extract. Fig 1 shows the process.

### CuO nanoparticles synthesis

CuO nanoparticles were effectively produced from *Punica granatum* leaves using a modified approach based on Vidovix, Quesada [7] method of synthesis. CuO nanoparticles were synthesized using a 0.2 M CuSO₄ ⋅ 5H₂O solution in 200 mL of deionized water. Then, the extract solution produced from the 100 g of the *Punica granatum* leaves was added dropwise to the copper sulfate solution under steady stirring at 80 °C for 15–20 minutes. To adjust the pH to 10, NaOH was added to the solution during the stirring process. After the reaction, the solution was dried in an oven at 60 °C for 24 hours. The resulting material was then calcined at 300 °C ± 10 °C for 2 hours in a muffle furnace to obtain CuO nanoparticles. The nanoparticles were cooled inside the furnace for over 24 hours before being used. The process is shown in Fig 1. The nanoparticle dosage range (0.25–1.0 wt. %) was selected based on previous reports showing that nanoparticle additions above 1 wt. % tend to cause agglomeration and reduce performance and its benefits in alkali-activated materials and cement materials [44,45]. Since lower dosages are observed to often yield minimal effect, 0.25–1.0 wt. % was the range chosen for the study to give the expected optimum results needed.

### Alkali-activated materials preparation

The alkali-activated materials were developed mainly from calcareous material found in Alevkaya, while fine aggregate was sourced from Beşparmak Mountain as crushed limestone. The Alevkaya calcareous material had over 90% calcium carbonate content, and the chemical composition was analyzed with X-ray fluorescence (XRF) spectroscopy, as shown in Table 1. The limestone was crushed to a standardized particle size of 2.36 mm as an inert material having a specific gravity of 2.67 and water absorption of 0.8%. The Alevkaya calcareous material was combined with crushed limestone at a weight ratio of 1:3 for the mixture.

**Table 1:** Chemical composition of alevkaya calcareous material.

Element	CaO	CO_2_	SiO_2_	Fe_2_O_3_	MgO	Al_2_O_3_	Na_2_O	SO_3_	Others
Result (%)	52.9	41.4	2.09	1.2	1.17	0.647	0.101	0.0794	0.3286

The alkali activator solution was prepared using sodium silicate (Na₂SiO₃, modulus of 2) from ZAG Kimya Ltd. and sodium hydroxide (NaOH, 10 M concentration) from Tekkim Ltd., maintaining an alkali activator to dry material ratio of 10% and a sodium silicate-to-sodium hydroxide ratio of 2.5:1. For samples containing CuO nanoparticles, the nanoparticles were first dispersed uniformly into the alkali activator solution, based on preliminary tests and previously reported methodologies [46,47]. The mix proportions are shown in Table 2.

**Table 2:** Mix proportions.

Mix ID	Calcareous Material	Crushed Limestone	Molding Pressure	CuO nanoparticles
	(w% %)	(w% %)	(MPa)	(w% %)
Control	25%	75%	15	0%
AAM + 0.25% CuO nanoparticles				0.25%
AAM + 0.5% CuO nanoparticles				0.50%
AAM + 1% CuO nanoparticles				1.00%

**Fig 2:**
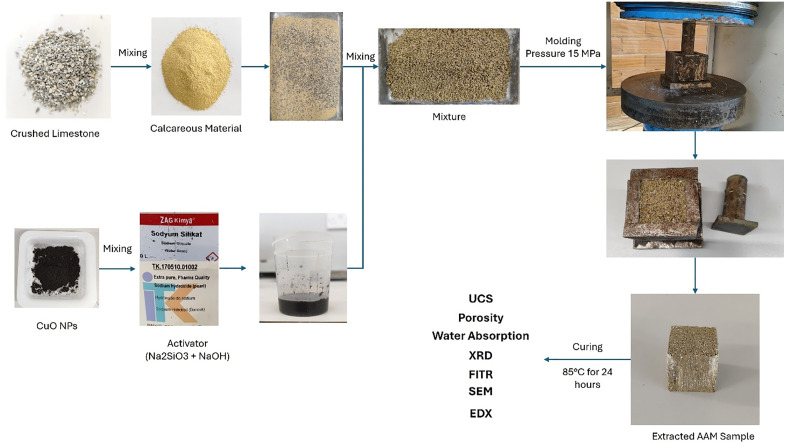
Alkali-activated material’s preparation procedure.


The prepared alkali activator suspension was subsequently introduced into the dry mix and thoroughly mixed until a homogeneous and semi-wet condition was reached, suitable for compaction. This approach helps reduce the total amount of alkali activator needed for the mix. After mixing, the material was poured into 5 cm × 5 cm × 5 cm cube molds, compacted at 15 MPa using a digital hydraulic compressor, and immediately extracted from the molds. Following compaction, the samples underwent curing at 85 °C for 24 hours, then continued curing at room temperature for an additional seven days. The expected reaction mechanism in calcareous material with sodium hydroxide and sodium silicate activators is shown in equations (1)–(5) [35]. The entire preparation and curing procedure is illustrated graphically in Fig 2.

$${\text{Na}}_{\text{2}}{\text{SiO}}_{\text{3}}+\text{3}{\text{H}}_{\text{2}}\text{O}\to \text{2}{\text{Na}}^{+}+\text{2}{\text{OH}}^{-}+{\text{Si}(\text{OH})}_{\text{4}}$$ (1)

$$\text{2}{\text{Na}}^{+}+{\text{CaCO}}_{\text{3}}+{\text{H}}_{\text{2}}\text{O}\to {\text{Ca}}^{\text{2}+}+{\text{Na}}_{\text{2}}{\text{CO}}_{\text{3}}.{\text{H}}_{\text{2}}\text{O}$$ (2)

$${\text{Ca}}^{\text{2}+}+{\text{Si}(\text{OH})}_{\text{4}}+{\text{H}}_{\text{2}}\text{O}\to \text{CSH gel}$$ (3)

$${\text{Ca}}^{\text{2}+}+{\text{Na}}^{+}+{\text{Si}(\text{OH})}_{\text{4}}+{\text{H}}_{\text{2}}\text{O}\to \text{CNSH gel}$$ (4)

$${\text{Si}(\text{OH})}_{\text{4}}\to \text{Silica gel}$$ (5)

### Characterization techniques

The CuO nanoparticles and the AAM samples produced were examined using various microscopic and spectroscopic methods. The Shimadzu (UV-2450) UV-visible spectrophotometer was used to measure the UV-visible spectrum of the nanoparticles, which was recorded between 200 and 800 nm. The produced CuO nanoparticles’ hydrodynamic (Z-average) size and Zeta potential were assessed using a Zeta sizer (Malvern Zetasizer pro lab), and the Malvern ZS nano software recorded the findings. The CuO nanoparticles and AAMs were analyzed using FTIR at frequencies between 4,000 and 450 cm^−1^ using a Shimadzu FT-IR (Prestige-21 Model Fourier transform spectrometer). The Rigaku (ZSX Primus II) X-ray diffractometer was used to investigate the crystalline structure over a 10° to 80° diffraction angle range for both CuO nanoparticles and the AAM. TEM was used to assess the morphology of the CuO nanoparticles, utilizing a JEOL 2100F High-Resolution Transmission Electron Microscope (HRTEM) at the METU Central Laboratory. The sample was prepared by dispersing the powdered specimen in ethanol and then subjected to ultrasonication for 30 minutes. Subsequently, one drop of the suspension was deposited onto a carbon-coated copper grid (CF200-Cu carbon film grid) using a micropipette and allowed to dry overnight. Particle dimensions were extracted from the TEM micrograph in ImageJ (scale from the 50 nm bar). Individual particles (n = 3, non-overlapping) were outlined manually; Feret’s diameter and area were recorded and used to compute the equivalent-circle diameter ($\text{ECD}=\text{2}*\sqrt{\frac{Area}{\pi }}$). Ellipse major/minor axes and aspect ratio were also obtained.

### Mechanical and microstructure testing

The compressive strength of the AAM samples was measured using a UTEST Multiplex Universal Electromechanics Testing Machine with a loading rate of 1 mm/min with a 1 N sensitivity load cell. The ASTM C20 was used to conduct porosity and water absorption tests to determine the percentage of voids and the amount of water absorbed by the AAM samples after immersion in water. SEM and EDS analyses were performed for AAM samples, using a QUANTA 400F Field Emission SEM at METU Central Laboratory. Before analysis, samples were crushed using a ball mill and sieved through a 45-micron sieve.

### Statistical analysis

One-way analysis of variance (ANOVA) was used to examine the impact of copper nanoparticles on each response variable (UCS, water absorption, and porosity) at various curing ages. The Dosage factor comprised four levels (0, 0.25, 0.50, 1.00%), with α = 0.05 (95% confidence interval). Upon obtaining significance from the omnibus ANOVA, pairwise comparisons were conducted utilizing Tukey’s HSD. The data are presented as mean ± SD (n = 3 for each level), accompanied by F(df₁, df₂) and adjusted p-values. The analyses were performed using OriginPro.

## Results and discussions

### UV–vis spectroscopy of CuO nanoparticles

**Fig 3:**
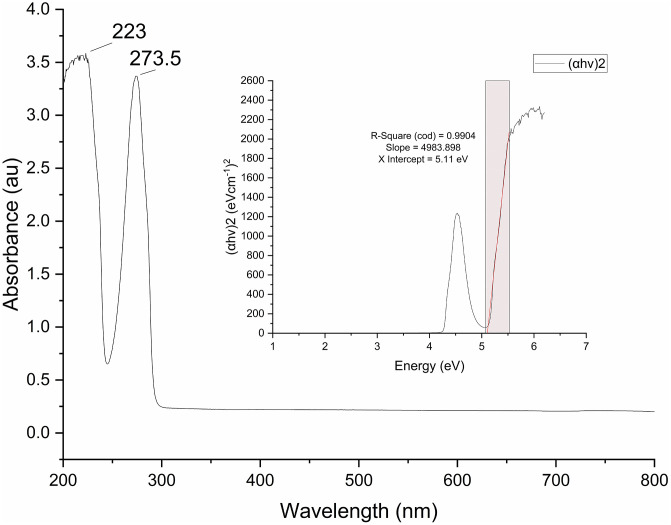
UV-visible absorption results of synthesized CuO nanoparticles using pomegranate leaf.

This method helps in suggesting the formation of nanoparticles by identifying a characteristic peak of a compound by how much light is absorbed. The CuO nanoparticles show two prominent absorption peaks at 223 nm and 273.5 nm in their UV-Vis spectrum, shown in Fig 3. These peaks are typical of CuO nanoparticles around this region. Depending on their size and synthesis conditions, CuO nanoparticles usually exhibit absorbance in the UV region, especially between 250 and 350 nm. The peak at 273.5 nm suggests that the creation of CuO nanoparticles aligns with the findings of Vidovix, Quesada [7], Rather and Sundarapandian [48], and Dutta, Kar [49]. Interactions with phytochemicals, such as flavonoids or phenolics, found in *Punica granatum* leaves, which exhibit unique UV absorption signatures, may contribute to the peak at 223 nm [50,51]. The UV–Vis absorption spectra exhibited characteristic features attributed to CuO nanoparticles; however, other residual phytochemicals from the *Punica granatum* extract may have also contributed to the observed absorption bands. The peaks from the extracted optical band gap may be slightly influenced due to the presence of these other phytochemicals, rather than showing only the pure CuO phase. Similar observations have been reported in other studies on green-synthesized metal oxide nanoparticles, where such residual capping molecules were observed to affect the optical properties from the UV-Vis spectra carried out [52–55].

Furthermore, Tauc analysis was conducted on the transmission data (Fig 3, inset) by converting absorbance to the absorption coefficient α = 2.303A/l (1 cm cuvette) and creating both direct (αhv)^2^ and indirect (αhv)^(1/2)^ plots. A linear segment was detected just in the UV range (about 5.1–5.6 eV); a least-squares regression of the direct plot produced R^2^ = 0.9904 and an x-intercept E_g_^Tauc^ = 5.11 eV. Due to CuO’s fundamental edge often residing in the visible range (about 1–2 eV) and the absence of a definite onset in that spectrum, the 5.11 eV value is understood as an apparent high-energy transition rather than the fundamental band gap. This result aligns with cautions in the literature that traditional Tauc extrapolation, initially designed for amorphous substances, may be misleading for highly crystalline materials [56]. The XRD suggests the presence of extremely crystalline CuO nanoparticles.

### Zeta size and zeta potential analysis of CuO nanoparticles

**Fig 4:**
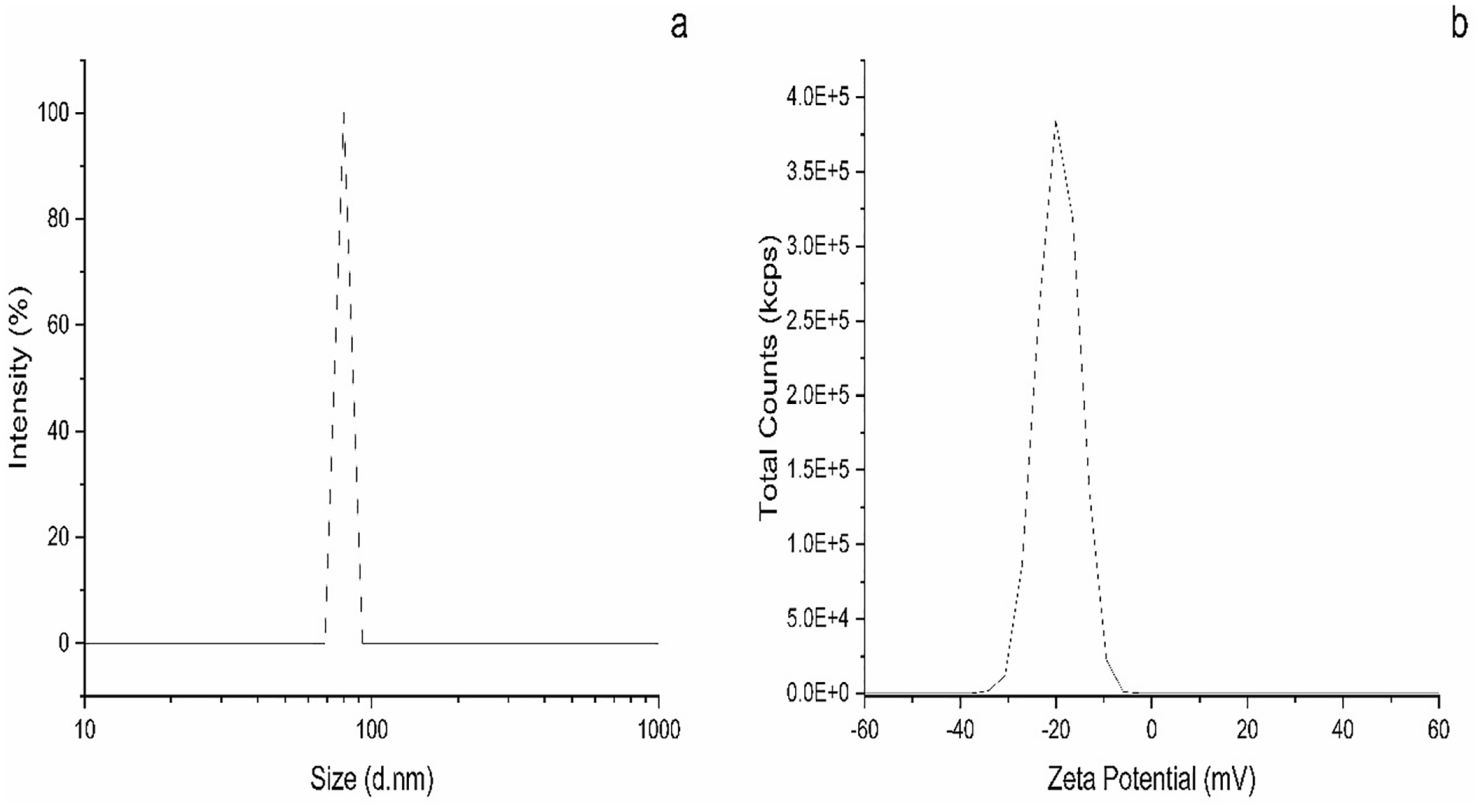
Synthesized CuO nanoparticles using pomegranate leaf a) mean size distribution, b) zeta potential.

The nanoparticles’ particle size distribution and surface charge stability were assessed using zeta size and potential studies. These investigations are essential for comprehending nanoparticle surface interactions, dispersion quality, and colloidal stability. As seen in Fig 4a, the DLS measurement of the zeta size revealed an average particle size of roughly 78 nm. A uniform particle size distribution is shown by the size distribution’s sharp peak, which represents a small size range. Because nanoparticles with a restricted size distribution often behave more consistently regarding mechanical reinforcement and interaction within a matrix, the CuO nanoparticles monodisperse nature is favorable. The 78 nm average size indicates that the green synthesis procedure effectively nucleated and controlled the growth of the CuO nanoparticles made with *Punica granatum* leaves. Additionally, the strong, distinct peak in the DLS results indicates no considerable agglomeration, suggesting that the size of the nanoparticles was maintained and further stabilized during the sintering process at 300°C. The surface charge of the CuO nanoparticles in a colloidal solution was determined using the zeta potential analysis, which is displayed in Fig 4b. The nanoparticles have a moderately negative surface charge, as indicated by the average zeta potential of −21 mV. Given that, particles with zeta potentials above ± 20 mV usually show sufficient electrostatic repulsion to avoid severe agglomeration, a zeta potential in this range indicates reasonable colloidal stability [57,58]. The zeta potential of the sintered synthetic CuO nanoparticles in this study suggests that the need for dispersion is important to enhance the effectiveness of the nanoparticles.

### Comparative analysis of the green-synthesized nanoparticles and chemogenic CuO nanoparticles on their size, crystallinity, and performance

#### Size and morphology.

The green-synthesized CuO nanoparticles in this study showed features of spherical morphology and narrow size distribution with minimal agglomeration. This can be suggested to be due to the natural capping action of phytochemicals within the leaf extract. This result, when compared to chemogenically synthesized CuO nanoparticles, which often show properties having a wider size distribution and greater agglomeration, which can be due to the use of harsh reducing agents and surfactants as reported by Dutta *et al.* (2015) in their study, where the hydrothermally synthesized CuO nanoparticles have sizes ranging from 50 to 100 nm with significant aggregation [49].

#### Crystallinity.

The XRD analysis confirmed the high crystallinity of the green-synthesized CuO nanoparticles, with distinct peaks corresponding to the monoclinic phase (JCPDS No. 48–1548). The green synthesis route resulted in minor amorphous content, likely due to organic residues, yet maintained high structural integrity. This is consistent with chemogenic methods reported by Vavouraki *et al*. (2021), where they used chemically synthesized CuO nanoparticles with similar crystallinity but noted impurities from residual precursors [44].

#### Performance in composite materials.

The incorporation of 0.5% green-synthesized CuO nanoparticles into AAMs showed a 37.3% increase in compressive strength and reduced porosity and water absorption. The green-synthesized nanoparticles also showed better dispersion at optimal concentrations (0.5%), whereas higher doses (1%) of chemogenic nanoparticles often lead to agglomeration and reduced effectiveness. This enhancement is comparable to that achieved with chemogenic CuO nanoparticles, as reported by Vavouraki et al. (2021) in their study [44].

#### Environmental and economic aspects.

The method of green synthesis avoids toxic chemicals, reduces energy consumption, and utilizes renewable resources, making it more sustainable than chemogenic methods. Studies such as those by Iravani (2011) and Vijayaram *et al.* (2024) emphasize the ecological benefits and cost-effectiveness of plant-mediated synthesis over conventional chemical routes [3,4].

#### FTIR of CuO nanoparticles.

**Fig 5:**
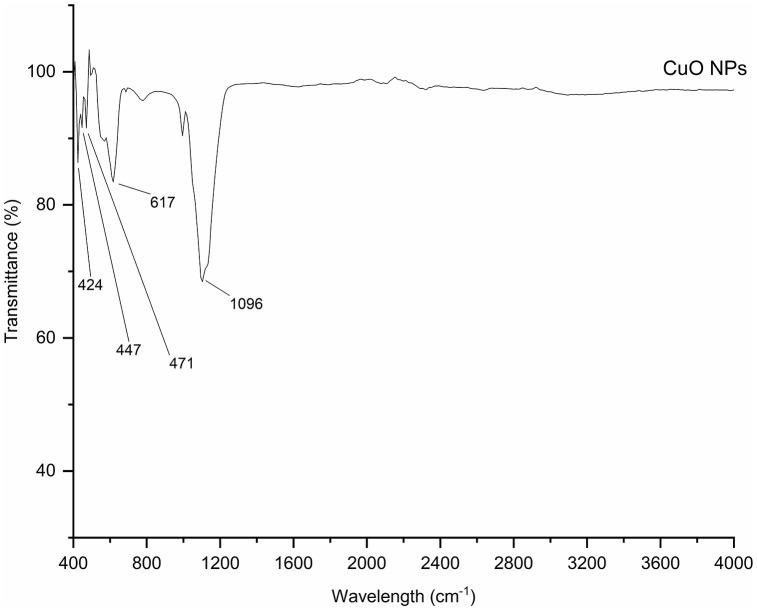
FTIR spectra of synthesized CuO nanoparticles using pomegranate leaf.

The FTIR spectroscopy analysis was performed to investigate the chemical bonds and functional groups in CuO nanoparticles synthesized using *Punica granatum* leaves, as presented in Fig 5. The Cu–O stretching vibration is responsible for a noticeable peak at 424 cm ⁻ ¹, which verifies the existence of CuO nanoparticles. This peak indicates that copper was successfully oxidized during the sintering process because it falls within the 400–500 cm ⁻ ¹ range typical for Cu–O bonds. Other bands at 447 cm ⁻ ¹, 470 cm ⁻ ¹, and 471 cm ⁻ ¹ also show metal-oxygen bonding, which is in line with CuO nanoparticles production, as other researchers mentioned similar peaks in their studies [59–62]. These metal oxide phases most likely crystallized more easily after sintering at 300°C, increasing the stability of the CuO nanoparticles.

Furthermore, as the nanoparticle dispersion underwent calcination before FTIR analysis, the spectra of the resultant CuO powder are anticipated to be mostly characterized by Cu–O lattice modes. In addition to the low-wavenumber Cu–O characteristics (∼400–500 cm ⁻ ¹) previously mentioned, a band about ∼617 cm ⁻ ¹ is commonly documented for tenorite-phase CuO and aligns with Cu–O stretching in crystalline CuO [63]. A broad band about ∼1096 cm ⁻ ¹ is most likely attributable to residual carbonaceous species on the surface post-incineration, typically associated with carbonate/oxygenated fragments that produce C–O stretching in the ∼1050–1100 cm ⁻ ¹ range, rather than intact phytochemical capping [64].

#### XRD of CuO nanoparticles.

**Fig 6:**
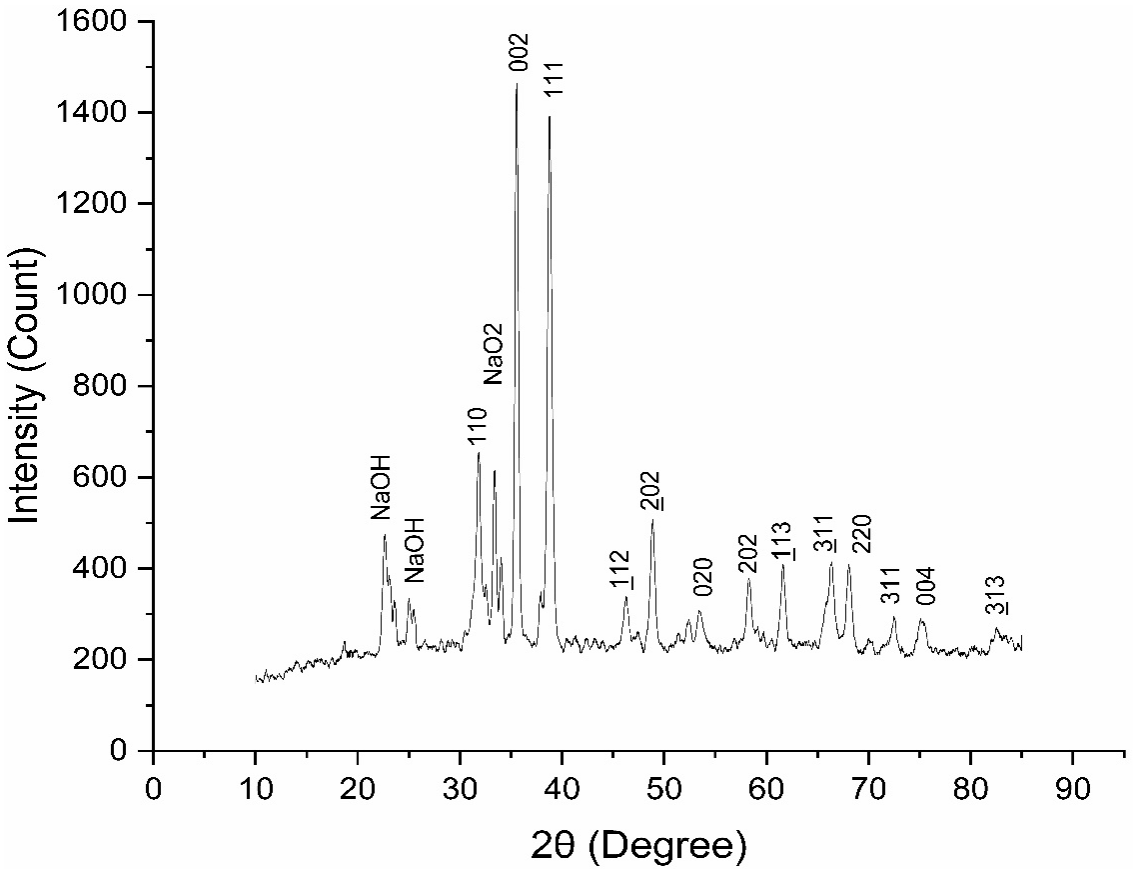
X.ray diffraction pattern of synthesized CuO nanoparticles using pomegranate leaf.

The XRD examination confirms the highly crystalline nature of the produced nanoparticles, which shows multiple distinctive diffraction peaks. Fig 6 displays the XRD pattern of the CuO nanoparticles synthesized with *Punica granatum* leaves. At 2θ values of roughly 32.5°, 35.5°, 38.7°, 48.7°, and 61.6° key diffraction peaks were seen. These can be attributed to the (110), (111), (002), (202), and (113) planes of CuO, respectively. These peaks are consistent with typical CuO diffraction patterns (JCPDS card No. 48–1548), suggesting that monoclinic copper oxide makes up the majority of the produced nanoparticles. These distinct peaks show that the 300 °C sintering procedure encouraged CuO crystallization, creating well-ordered copper oxide structures. One of the strongest reflections for copper oxide is the (111) plane, which is shown by the peak at 2θ = 38.7°. This implies that the CuO nanoparticles have a high degree of crystallinity, which is frequently preferred for applications requiring stable and well-structured materials. Since broadening of diffraction peaks is generally linked to lower particle sizes, the sharpness of the peaks also suggests that the nanoparticles are highly crystalline and relatively small [65].

Apart from CuO, the existence of faint peaks at lower 2θ values, like at 20° and 30°, can be the result of leftover substances from the synthesis process, like NaOH, but they have little effect on the structure of the CuO nanoparticles. The lack of metallic Cu peaks indicates that the 300 °C sintering produced CuO nanoparticles by completely oxidizing copper.

#### TEM of CuO nanoparticles.

**Fig 7:**
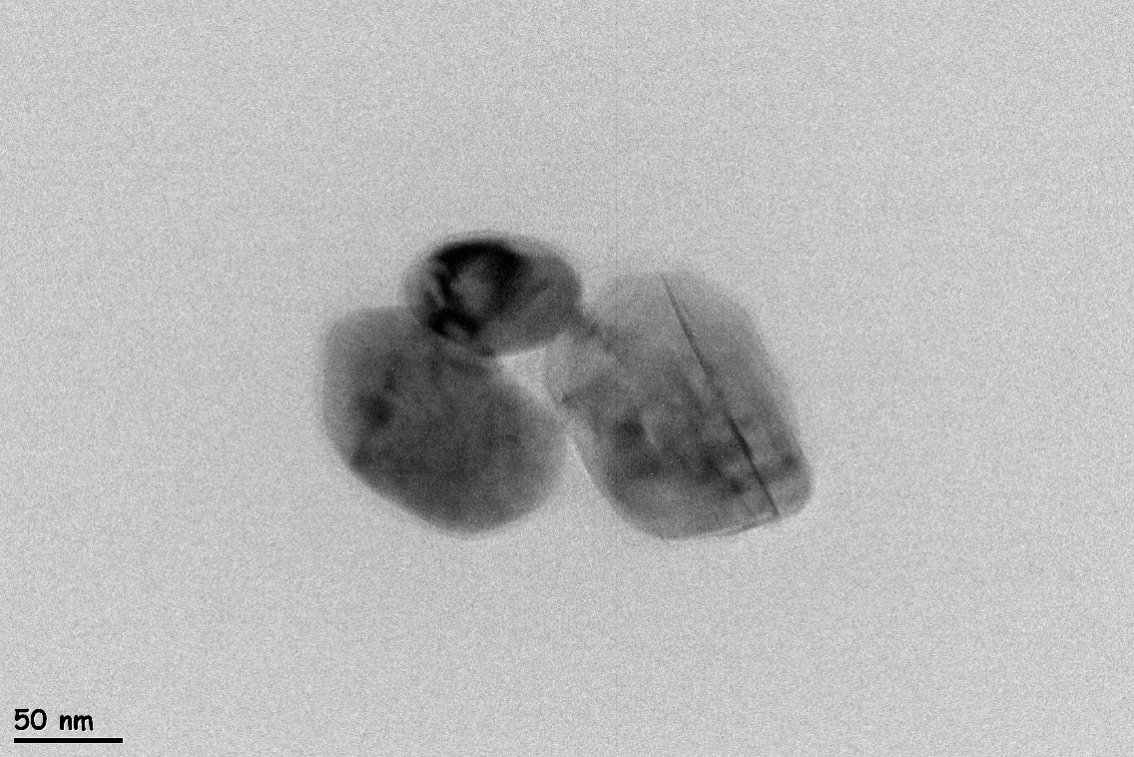
TEM micrograph showing the morphology and particle size of synthesized CuO nanoparticles.

TEM was employed to analyze the morphology of the synthesized CuO nanoparticles. The representative micrograph (Fig 7) illustrates faceted, near-spherical crystallites exhibiting modest anisotropy and some agglomeration, which can be attributed to the drying and preparation processes. Manual measurements using ImageJ from the available field (n = 3) produced Feret diameters of 81.8, 128.5, and 140.6 nm (mean 117.0 nm, range 81.8–140.6 nm) and equivalent-circle diameters (ECD) of 66.6, 103.3, and 118.4 nm (mean 96.1 nm), as detailed in Table 3. Due to the availability of only a single field, these values are presented as per-particle measurements instead of a complete size distribution. The DLS hydrodynamic diameter of 78 nm in water is not anticipated to correspond directly with TEM core sizes, as DLS assesses solvated or ensemble dimensions, while TEM evaluates dry particle cores. The TEM image validates the presence of nanoscale CuO particles exhibiting distinct boundaries, indicating successful nanoparticle synthesis [66,67].

**Table 3:** TEM derived particle dimensions from a single micrograph; values in nanometers (nm).

Particle	Feret diameter†	ECD‡	Major (ellipse)	Minor (ellipse)	Aspect ratio (Major/Minor)	Circularity	Solidity
1	81.758	66.58	81.069	54.667	1.483	0.674	1
2	128.474	103.3	126.916	84.091	1.509	0.663	0.999
3	140.617	118.5	136.659	102.652	1.331	0.751	0.997
Mean	116.95	96.12	114.88	80.47	1.441	—	—

†Feret diameter = longest caliper across the particle (largest chord).

‡ $\text{ECD}(\text{equivalent}-\text{circle diameter}=\text{2}\sqrt{A/\pi }$, where *A* is the outlined area.

**Fig 8:**
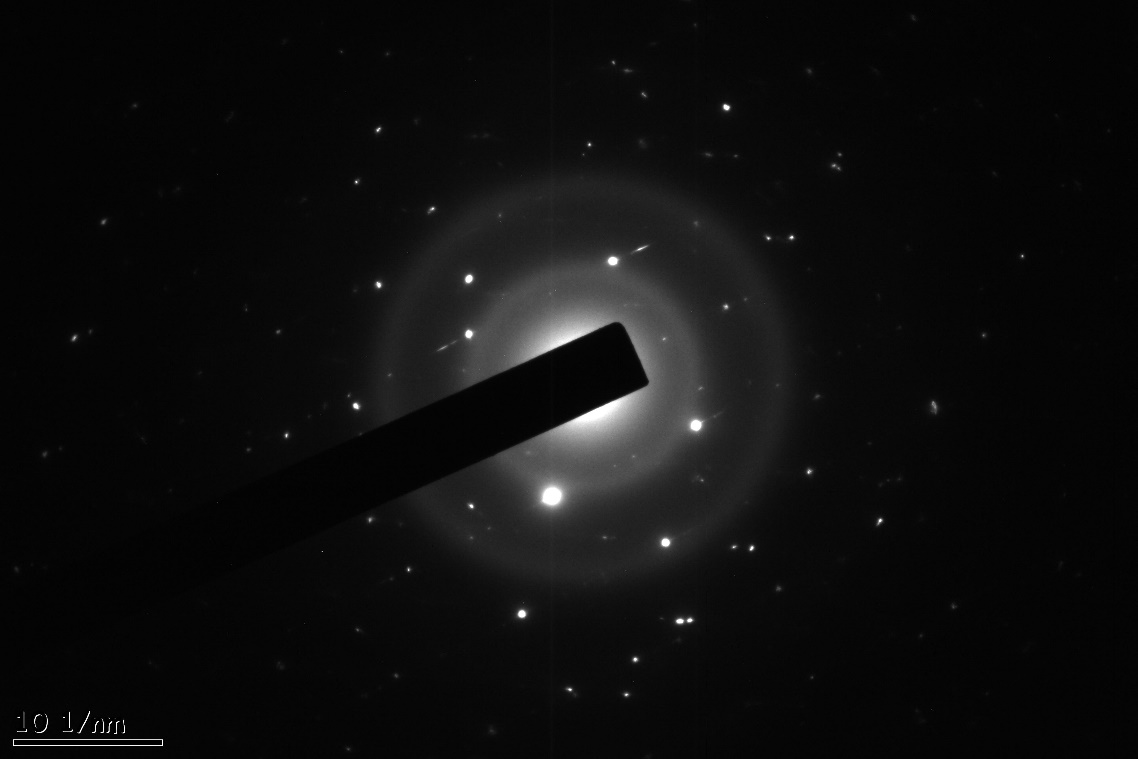
SAED pattern of synthesized CuO nanoparticles.

Distinct diffraction rings, along with bright spots, are indicative of the polycrystalline nature of the CuO nanoparticles. This is observable in the Selected Area Electron Diffraction (SAED) pattern (Y), which can be found in Fig 8. Each diffraction ring manifests specific crystallographic planes, confirming good crystallinity. Multiple discrete diffraction spots were also observed, affirming that the nanoparticles have well-defined crystal structures. The results obtained through SAED agreed with the XRD analysis, confirming the crystalline characteristics of the CuO nanoparticles developed from *Punica granatum* leaves after thermal treatments.

### Application of the CuO nanoparticles with AAMs and the characterization of their results

#### XRD of AAMs with CuO nanoparticles.

**Fig 9:**
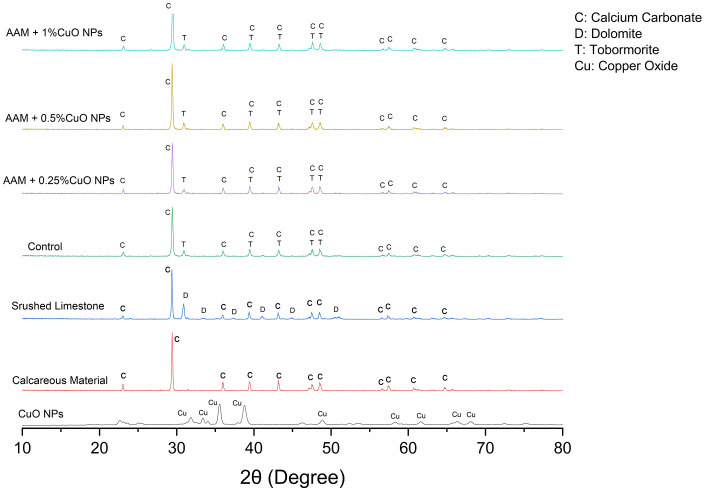
X.ray diffraction pattern of raw materials and different AAMs mixes.

The XRD analysis was performed on AAMs with CuO nanoparticles to determine their crystallinity, phase identification, and lattice parameters. Fig 9 displays the XRD patterns of crushed limestone, calcareous material, and the synthesized CuO nanoparticles, in addition to the patterns of AAMs containing different concentrations of CuO nanoparticles. The substantial calcium carbonate concentration in the raw materials is confirmed by the noticeable peaks in the crushed limestone’s XRD pattern linked to dolomite (D) and calcium carbonate (C). The calcareous material pattern also shows prominent calcium carbonate phases in line with its composition. The XRD patterns of AAM samples containing 0.25%, 0.5%, and 1% CuO nanoparticles exhibit clear reflections for calcium carbonate (C) alongside a broad amorphous hump indicative of a tobermorite-like C-(A)-S-H gel, rather than separate crystalline tobermorite peaks. The continuous existence of this gel in all mixtures indicates that alkali activation generated the necessary binding phase linked to strength enhancement in AAMs. The CuO peaks observed in the produced CuO nanoparticles are less prominent in the AAM diffractograms, perhaps due to the prevailing carbonate reflections and the gel hump diminishing their visibility. Consequently, even in the presence of copper oxides, they are challenging to discern in the composite’s diffraction pattern. The AAM absent of CuO nanoparticles (Control) exhibits pronounced carbonate reflections and the same gel hump, akin to the mixtures containing CuO. This consistency indicates that the creation of the crucial binding C-(A)-S-H gel is not impeded by the incorporation of CuO nanoparticles.

#### FTIR of AAMs with CuO nanoparticles.

**Fig 10:**
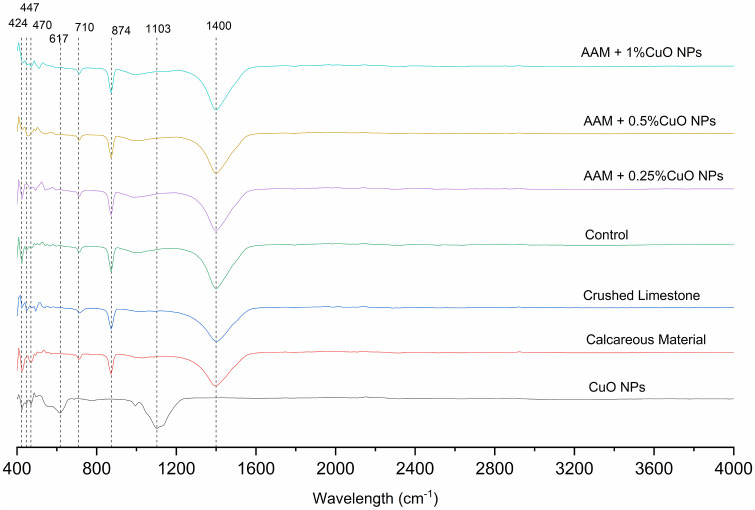
FTIR pattern of raw materials and AAMs mixes.

The FTIR spectroscopy analysis was performed to investigate the chemical bonds and functional groups in AAMs with CuO nanoparticles. Fig 10 illustrates the FTIR spectroscopy investigation of AAMs with different CuO nanoparticle concentrations. The FTIR spectra have distinctive peaks in the 400–1600 cm ⁻ ¹ range. The successful incorporation of CuO nanoparticles into the AAM is confirmed by the peaks between 424 cm ⁻ ¹ and 471 cm ⁻ ¹, which are ascribed to the Cu–O stretching vibrations. These peaks become more noticeable as the concentration of CuO nanoparticles rises. The AAM with 1% CuO nanoparticles has the maximum intensity, suggesting a higher presence of copper oxide.

The peaks at 710 cm ⁻ ¹ and 874 cm ⁻ ¹ indicate the calcium carbonate (CaCO₃) phases, which are derived from the broken limestone and calcareous materials included in the formulation [33,36,37,39–41]. These peaks shown are true for every sample, suggesting that the carbonate phases in the AAM matrix are not disturbed by adding CuO nanoparticles.

In keeping with the calcium carbonate phases found in the material, a peak at 1400 cm ⁻ ¹ is also seen, which corresponds to carbonate ion (CO₃²⁻) stretching. When CuO nanoparticles are added, the intensity of this peak stays the same, indicating that the presence of the nanoparticles does not affect the carbonate phases.

#### SEM/EDS of AAM mixes.

**Fig 11:**
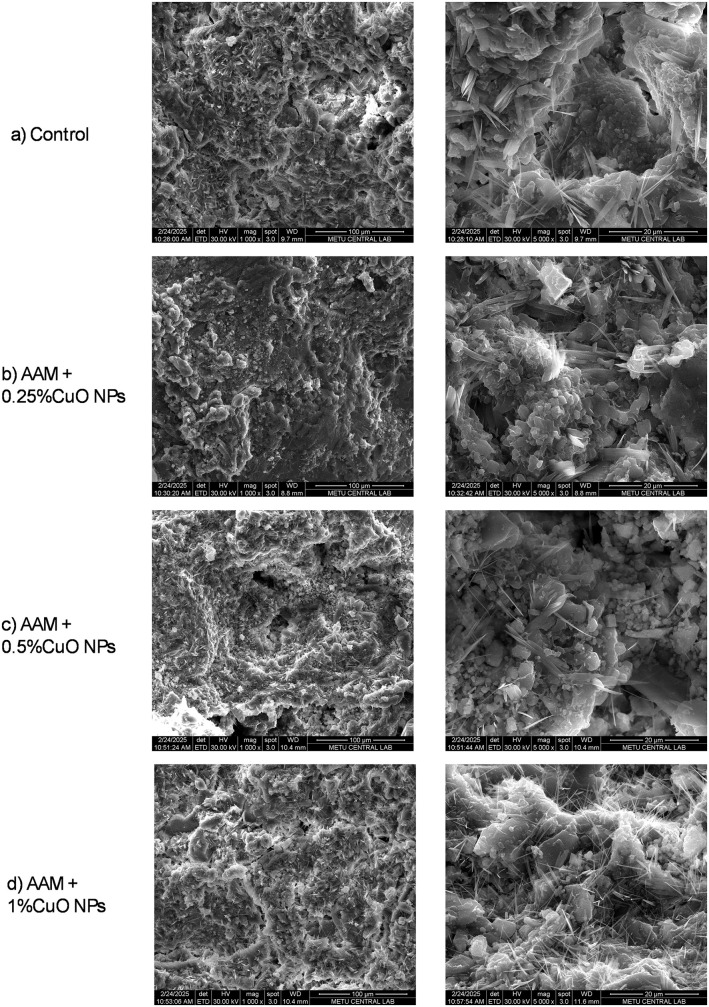
Scanning electron microscope image of the AAM mixes.

**Fig 12:**
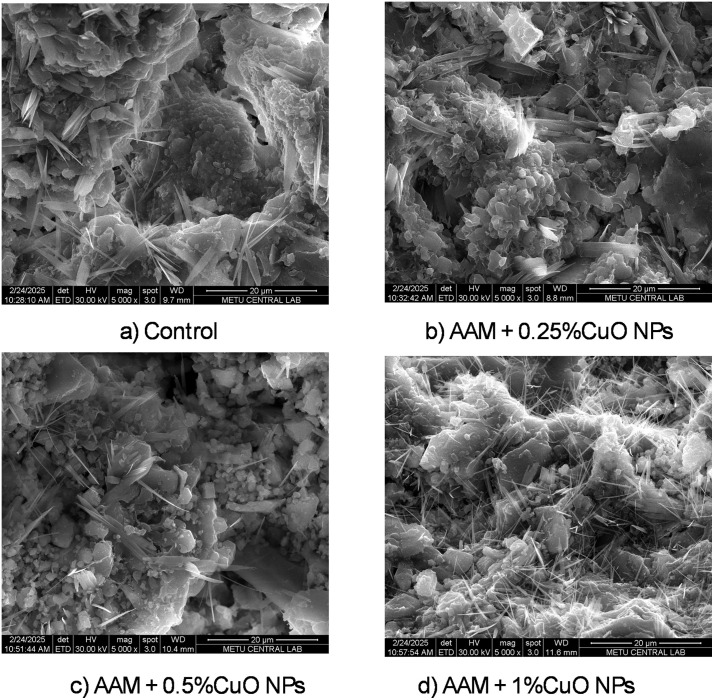
SEM showing the formation of needle-shaped crystals.

Fig 11 presents SEM images of the AAM mixtures at seven days. The overall shape is consistent throughout mixtures, with enhanced compactness noted in specimens containing copper oxide nanoparticles. The elevated surface area of the nanoparticles shown in the micrographs can facilitate further nucleation and development of the alkali-activated binder. Crystalline hydrate characteristics are evident in all mixtures (Fig 12); portlandite, when present, is recognized by its distinctive XRD reflections rather than appearance alone. An increased apparent density of these acicular structures is observed in the AAM + 1% CuO nanoparticles mixture. At higher dosages, nanoparticle aggregation may result in localized variation in feature distribution, potentially diminishing their efficacy in reinforcing the structure.

**Fig 13:**
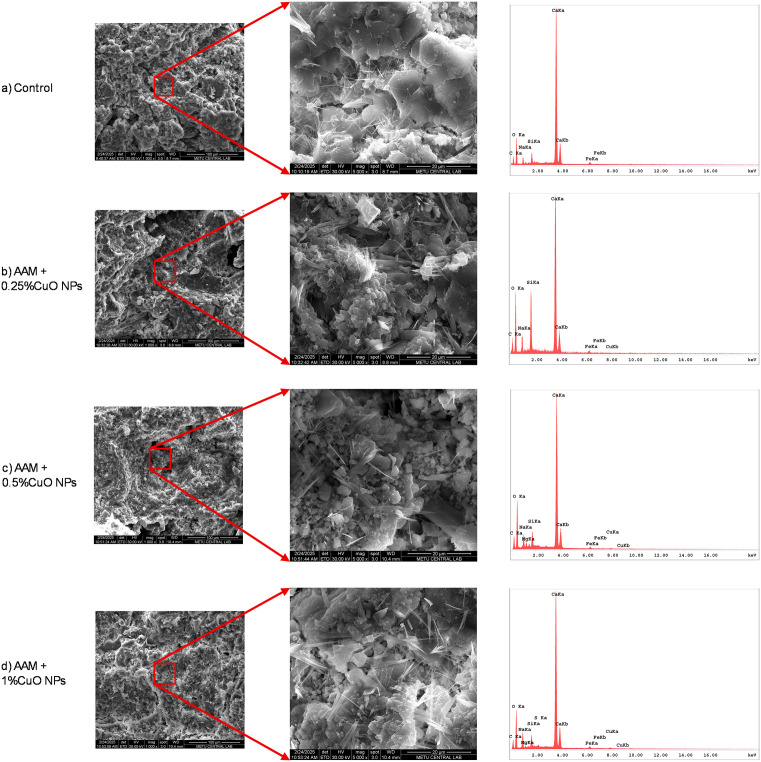
EDS analysis of AAM mixes.

EDS analysis was also performed to identify the chemical elements present in the AAMs. The elemental compositions of the AAMs with and without CuO nanoparticles are shown in Fig 13. The EDS results indicate the presence of C, O, Na, Mg, Si, Ca, Fe, and Cu in the mixes, as presented in Table 4. The presence of Cu in the mixes containing 0.25% and 0.5% CuO nanoparticles (measured as 0.12% and 0.34%, respectively) is consistent with the initial amounts of CuO added. The values reported in the table represent the Cu element only, excluding oxygen. In the mix containing 1% CuO nanoparticles, the EDS result shows 1.02% Cu in the analyzed area, slightly exceeding the added amount. This suggests a non-homogeneous dispersion of CuO nanoparticles, which may reduce their overall effectiveness.

**Table 4:** Elemental composition for AAM mixes from EDS analysis.

Sample	Control		AAM + 0.25%CuO NPs		AAM + 0.5%CuO NPs		AAM + 1%CuO NPs	
Element	Wt %	At %	Wt %	At %	Wt %	At %	Wt %	At %
C K	14.89	24.64	20.18	30.55	18.74	28.35	16.21	25.21
O K	42.02	52.19	43.57	49.5	47.02	53.41	44.87	52.39
NaK	3.92	3.39	5.96	4.71	5.42	4.28	5.88	4.78
MgK	----	----	----	----	1.66	1.24	3.69	2.84
SiK	2.48	1.76	8.21	5.32	2.72	1.76	7.65	5.09
CaK	35.43	17.57	21.08	9.56	23.53	10.67	18.88	8.8
FeK	1.26	0.45	0.88	0.29	0.54	0.18	1.79	0.6
CuK	----	----	0.12	0.34	0.36	0.1	1.02	0.3
Total	100	100	100	100	100	100	100	100

#### UCS test of the AAMs with CuO nanoparticles.

**Fig 14:**
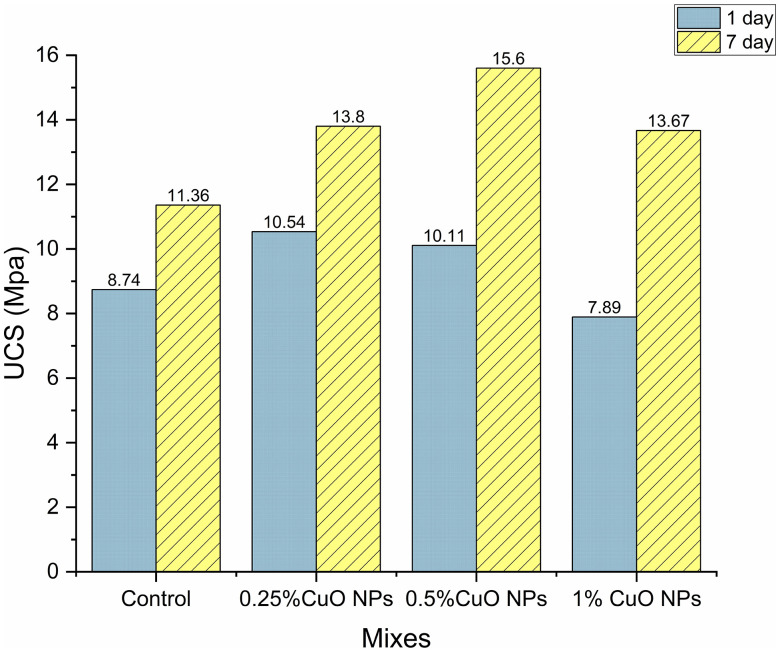
Unconfined compressive strength of AAM mixes.

After one and seven days of curing, the mechanical performance of AAMs with varying concentrations of CuO nanoparticles was evaluated using UCS tests, as shown in Fig 14. The findings shed light on how CuO nanoparticles impact the AAM matrix’s strength development.

Adding CuO nanoparticles was observed to improve compressive strength, according to the UCS values after one day of curing. When 0.25% CuO nanoparticles were added, the strength rose by around 20% compared to the AAM without CuO nanoparticles, which acts as a control. In contrast to the control, the strength increase was 15.6% when the CuO nanoparticle level was increased to 0.5%. Higher CuO nanoparticle concentrations do not appear to significantly contribute to incremental strength improvements, as seen by the compressive strength not improving when the CuO nanoparticle content was raised to 1%. This may be because it is challenging to maintain consistent dispersion of nanoparticles at larger concentrations. Tables 5–7 present the descriptive statistics, one-way ANOVA, and Tukey HSD results, respectively, for the 1^st^ day UCS. One-way ANOVA revealed a significant dosage effect, F (3, 8) = 540.0, p < 0.0001, η² = 0.995. Tukey’s HSD showed that 0.25% and 0.50% were both higher than the control (mean differences of 1.794 and 1.362 MPa; both p < 0.0001). It also showed that 0.25% was higher than 0.50% (diff 0.432 MPa; p = 0.0018), and that 1.00% was lower than all other levels (e.g., vs 0.25%: diff −2.645 MPa; p < 0.0001).

**Table 5:** 1^st^ Day UCS descriptive statistics.

CuO NPs (%)	N	Mean	Standard Deviation	SE of Mean
Control	3	8.7508	0.08984	0.05187
0.25	3	10.54507	0.14254	0.08229
0.5	3	10.11267	0.05583	0.03223
1	3	7.89987	0.04003	0.02311

**Table 6:** 1^st^ Day UCS one-way ANOVA results.

Source	DF	Sum of Squares	Mean Square	F Value	P Value
Model	3	13.40902	4.46967	540.00097	<0.0001
Error	8	0.06622	0.00828		
Total	11	13.47524			

**Table 7:** 1^st^ Day UCS Tukey test result.

	MeanDiff	SEM	q Value	Prob	Alpha	Sig	LCL	UCL
0.25 Control	1.794	0.074	34.159	<0.0001	0.05	1	1.556	2.032
0.5 Control	1.362	0.074	25.927	<0.0001	0.05	1	1.124	1.600
0.5 0.25	−0.432	0.074	8.232	0.00177	0.05	1	−0.670	−0.195
1 Control	−0.851	0.074	16.200	<0.0001	0.05	1	−1.089	−0.613
1 0.25	−2.645	0.074	50.359	<0.0001	0.05	1	−2.883	−2.407
1 0.5	−2.213	0.074	42.127	<0.0001	0.05	1	−2.451	−1.975

All samples’ UCS results show further improvement after seven days of curing. Because of the ongoing alkali activation, the AAM without CuO nanoparticles demonstrated a 30% strength increase. Nevertheless, the compressive strength rose 54% with 0.5% CuO nanoparticles compared to the first day, showing the continuous beneficial effect of CuO nanoparticles on matrix densification and forming a more robust internal structure, resulting in 37.3% more compressive strength than the control mix. Although the 1% CuO nanoparticles sample’s UCS value was 73% higher than the 1^st^ day strength, it was still less than the 0.5% CuO nanoparticles sample’s results.

Tables 8–10 present the descriptive statistics, one-way ANOVA, and Tukey HSD results, respectively for the 7^th^ day UCS. The one-way ANOVA revealed a significant dose effect, with F(3, 8) = 70.66, p < 0.0001, and η² = 0.964. The mean values (MPa) for the groups are as follows: control = 11.36, 0.25% = 13.85, 0.50% = 15.60, 1.00% = 13.67. Tukey’s HSD indicated that both 0.25% and 0.50% significantly exceeded the control (differences of 2.49 [95% CI 1.55, 3.43], p = 0.00013; 4.24 [3.30, 5.18], p < 0.0001), with 0.50% surpassing 0.25% (difference of 1.75 [0.81, 2.69], p = 0.00149). Additionally, 1.00% was greater than the control (difference of 2.31 [1.37, 3.25], p = 0.00022), but did not exceed 0.25% (difference of −0.18, p = 0.924), and was lower than 0.50% (difference of −1.93 [−2.87, −0.99], p = 0.00077). Generally, strength increases with dosage up to 0.50% (the peak), while 1.00% does not surpass 0.25% and is less effective than 0.50%.

**Table 8:** 7^th^ Day UCS descriptive statistics.

CuO NPs (%)	N Analysis	N Missing	Mean	Standard Deviation	SE of Mean
Control	3	0	11.36	0.16406	0.09472
0.25	3	0	13.85	0.38379	0.22158
0.5	3	0	15.6	0.52468	0.30292
1	3	0	13.67	0.2544	0.14688

**Table 9:** 7^th^ Day UCS one-way ANOVA results.

Source	DF	Sum of Squares	Mean Square	F Value	P Value
Model	3	27.2502	9.0834	70.65822	<0.0001
Error	8	1.02843	0.12855		
Total	11	28.27863			

**Table 10:** 7^th^ Day UCS Tukey test result.

Comparison	MeanDiff	SEM	q Value	Prob	Alpha	Sig	LCL	UCL
0.25 Control	2.49	0.293	12.029	0.00013	0.05	1	1.553	3.427
0.5 Control	4.24	0.293	20.483	<0.0001	0.05	1	3.303	5.177
0.5 0.25	1.75	0.293	8.454	0.00149	0.05	1	0.813	2.687
1 Control	2.31	0.293	11.159	0.00022	0.05	1	1.373	3.247
1 0.25	−0.18	0.293	0.870	0.92449	0.05	0	−1.117	0.757
1 0.5	−1.93	0.293	9.323	0.00077	0.05	1	−2.867	−0.993

**Fig 15:**
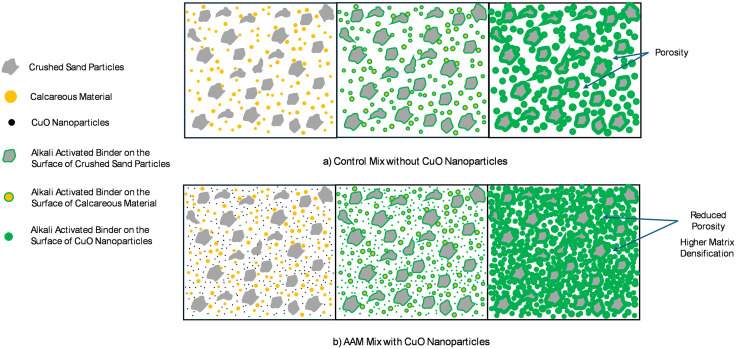
Mechanism of CuO nanoparticle enhancement in AAM microstructure.

CuO nanoparticles performed best at a concentration of 0.5%, where they are evenly distributed and efficiently support matrix densification and strength improvement. This result is parallel to the study of Vavouraki, Gounaki [44] where a stronger material is produced at this concentration because CuO nanoparticles most likely serve as nucleation sites for synthesizing calcium silicate hydrate (C–S–H), the mechanism is illustrated in Fig 15. However, the strength does not improve proportionately over 1% CuO nanoparticles, mostly because of decreased particle dispersion efficiency, which restricts additional matrix reinforcement.

#### Porosity and water absorption.

**Fig 16:**
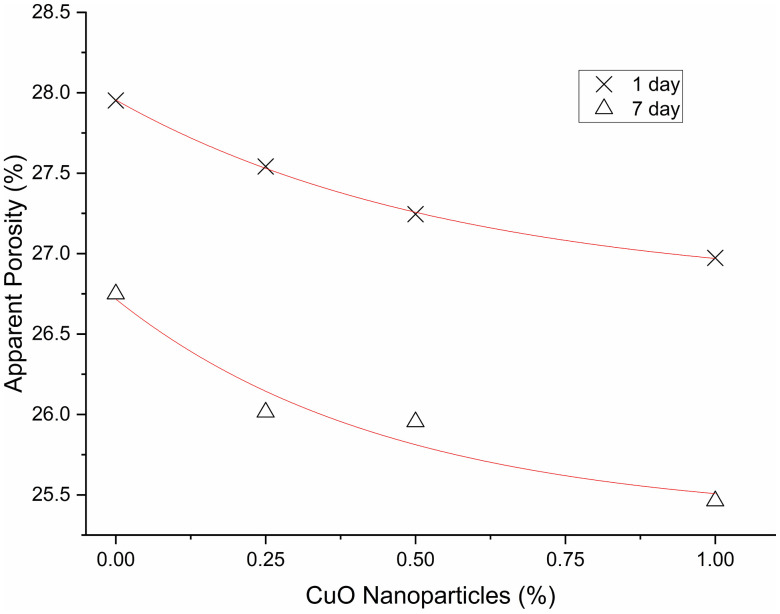
Porosity of AAM mixes.

Tables 11–13 display the 1-day descriptive statistics, one-way ANOVA, and Tukey HSD, while Tables 14–16 exhibit the similar 7-day results. Apparent porosity reduces consistently with increasing CuO nanoparticles dosage at both time points (1-day averages: 0% = 0.2795, 0.25% = 0.2754, 0.50% = 0.2725, 1.00% = 0.2697; 7-day averages: 0% = 0.2675, 0.25% = 0.2601, 0.50% = 0.2595, 1.00% = 0.2546), aligning with the gradual development of gel during alkali activation. Nonetheless, the 1-day ANOVA revealed a significant overall effect (F(3,8) = 7.12, p = 0.01198), with Tukey’s test indicating that only the 1.00% mixture was significantly lower than the control (Δ = −0.0098; 95% CI −0.0168 to −0.0027; adjusted p = 0.0097), but the comparison between the 0.50% mixture and the control did not achieve significance (adjusted p = 0.053). At 7 days, the overall test yielded no significance (F(3,8) = 2.09, p = 0.180), and no pairwise differences were identified. Consequently, porosity decreases with dosage as shown in Fig 16; nonetheless, statistically significant reductions are confined to 1.00% at 1 day, with no verified differences across dosages at 7 days.

**Table 11:** 1^st^ Day porosity descriptive statistics.

CuO NPs (%)	N Analysis	N Missing	Mean	Standard Deviation	SE of Mean
Control	3	0	0.27946	0.00373	0.00215
0.25	3	0	0.27543	9.30E-04	5.37E-04
0.5	3	0	0.27247	0.00116	6.69E-04
1	3	0	0.26971	0.00364	0.0021

**Table 12:** 1^st^ Day porosity one-way ANOVA results.

Source	DF	Sum of Squares	Mean Square	F Value	P Value
Model	3	1.57E-04	5.23E-05	7.12334	0.01198
Error	8	5.87E-05	7.34E-06		
Total	11	2.16E-04			

**Table 13:** 1^st^ Day porosity tukey test result.

Comparison	MeanDiff	SEM	q Value	Prob	Alpha	Sig	LCL	UCL
0.25 Control	−0.004	0.002	2.576	0.331	0.05	0	−0.0111	0.0031
0.5 Control	−0.007	0.002	4.466	0.053	0.05	0	−0.0141	0.0001
0.5 0.25	−0.003	0.002	1.890	0.568	0.05	0	−0.0100	0.0041
1 Control	−0.010	0.002	6.232	0.010	0.05	1	−0.0168	−0.0027
1 0.25	−0.006	0.002	3.656	0.119	0.05	0	−0.0128	0.0014
1 0.5	−0.003	0.002	1.767	0.616	0.05	0	−0.0099	0.0043

**Table 14:** 7^th^ Day porosity descriptive statistics.

CuO NPs (%)	N Analysis	N Missing	Mean	Standard Deviation	SE of Mean
Control	3	0	0.26753	0.00452	0.00261
0.25	3	0	0.2601	0.00834	0.00481
0.5	3	0	0.25946	0.00795	0.00459
1	3	0	0.25464	0.00309	0.00179

**Table 15:** 7^th^ day porosity one-way ANOVA results.

Source	DF	Sum of Squares	Mean Square	F Value	P Value
Model	3	2.55E-04	8.50E-05	2.08908	0.18007
Error	8	3.26E-04	4.07E-05		
Total	11	5.81E-04			

**Table 16:** 7^th^ Day porosity tukey test result.

Comparison	MeanDiff	SEM	q Value	Prob	Alpha	Sig	LCL	UCL
0.25 Control	−0.007	0.005	2.018	0.519	0.05	0	−0.0241	0.0093
0.5 Control	−0.008	0.005	2.192	0.455	0.05	0	−0.0248	0.0086
0.5 0.25	−0.001	0.005	0.174	0.999	0.05	0	−0.0173	0.0160
1 Control	−0.013	0.005	3.500	0.139	0.05	0	−0.0296	0.0038
1 0.25	−0.005	0.005	1.483	0.728	0.05	0	−0.0221	0.0112
1 0.5	−0.005	0.005	1.308	0.793	0.05	0	−0.0215	0.0119

**Fig 17:**
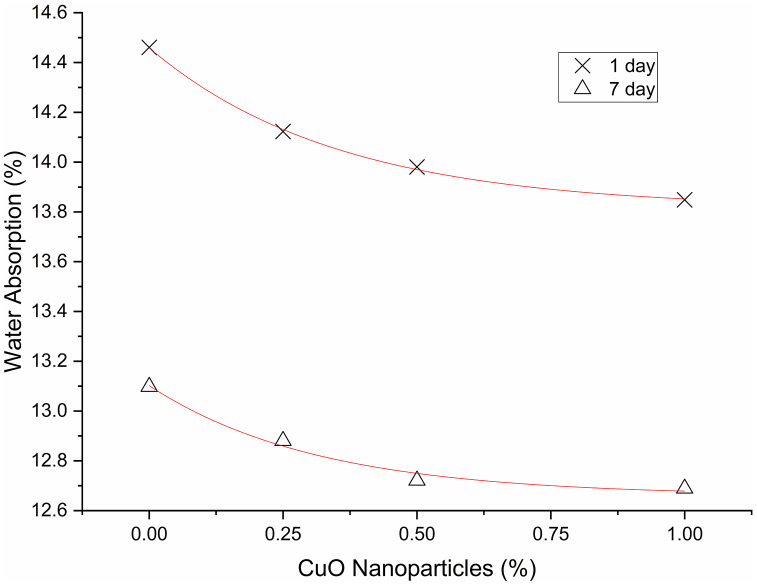
Water absorption of AAM mixes.

Tables 17–19 provide the 1-day descriptive statistics, one-way ANOVA, and Tukey HSD; Tables 20 and 21 present the corresponding 7-day results. Water absorption exhibits a decreasing trend with increasing CuO nanoparticles dosage at both ages. For the 1-day metrics: 0% corresponds to 14.46%, 0.25% to 14.12%, 0.50% to 13.98%, and 1.00% to 13.85%. For the 7-day metrics: 0% is 13.10%, 0.25% is 12.88%, 0.50% is 12.73%, and 1.00% is 12.69%. On day 1, the overall ANOVA yielded significant results, F(3,8) = 6.59, p = 0.0149. Tukey’s test revealed that 0.50% and 1.00% were significantly lower than the control, with differences of −0.00477 and −0.00602 in fraction units, approximately translating to −0.48 and −0.60 percentage points, respectively (adjusted p = 0.041 and 0.0126). Other pairwise comparisons did not reach significance. Over a period of 7 days, the ANOVA results indicated no significance, F(3,8) = 0.635, p = 0.613, and no Tukey pairwise differences were identified. Consequently, absorption generally diminishes with increased dosage as shown in Fig 17; however, statistically significant reductions are limited to 0.50–1.00% at 1 day Table 22, with no verified dosage effects observed at 7 days.

**Table 17:** 1^st^ Day water absorption descriptive statistics.

CuO NPs (%)	N Analysis	N Missing	Mean	Standard Deviation	SE of Mean
Control	3	0	0.1446	0.0026	0.0015
0.25	3	0	0.1412	0.0019	0.0011
0.5	3	0	0.1398	0.0002	0.0001
1	3	0	0.1385	0.0014	0.0008

**Table 18:** 1^st^ day water absorption one-way ANOVA results.

Source	DF	Sum of Squares	Mean Square	F Value	P Value
Model	3	6.06E-05	2.02E-05	6.59017	0.01486
Error	8	2.45E-05	3.07E-06		
Total	11	8.52E-05			

**Table 19:** 1^st^ Day water absorption tukey test result.

Comparison	MeanDiff	SEM	q Value	Prob	Alpha	Sig	LCL	UCL
0.25 Control	−0.0034	0.0014	3.334	0.164	0.05	0	−0.0080	0.0012
0.5 Control	−0.0048	0.0014	4.720	0.041	0.05	1	−0.0094	−0.0002
0.5 0.25	−0.0014	0.0014	1.386	0.765	0.05	0	−0.0060	0.0032
1 Control	−0.0060	0.0014	5.951	0.013	0.05	1	−0.0106	−0.0014
1 0.25	−0.0027	0.0014	2.616	0.319	0.05	0	−0.0072	0.0019
1 0.5	−0.0012	0.0014	1.231	0.820	0.05	0	−0.0058	0.0033

**Table 20:** 7^th^ day water absorption descriptive statistics.

CuO NPs (%)	N Analysis	N Missing	Mean	Standard Deviation	SE of Mean
Control	3	0	0.13102	0.00211	0.00122
0.25	3	0	0.12878	0.00612	0.00353
0.5	3	0	0.12734	0.00344	0.00199
1	3	0	0.12689	0.00340	0.00196

**Table 21:** 7^th^ Day water absorption one-way ANOVA results.

Source	DF	Sum of Squares	Mean Square	F Value	P Value
Model	3	3.11E-05	1.04E-05	0.63471	0.61326
Error	8	1.31E-04	1.63E-05		
Total	11	1.62E-04			

**Table 22:** 7^th^ Day water absorption tukey test result.

Comparison	MeanDiff	SEM	q Value	Prob	Alpha	Sig	LCL	UCL
0.25 Control	−0.0022	0.0033	0.9590	0.9025	0.05	0	−0.013	0.008
0.5 Control	−0.0037	0.0033	1.5766	0.6912	0.05	0	−0.014	0.007
0.5 0.25	−0.0014	0.0033	0.6176	0.9704	0.05	0	−0.012	0.009
1 Control	−0.0041	0.0033	1.7703	0.6146	0.05	0	−0.015	0.006
1 0.25	−0.0019	0.0033	0.8113	0.9372	0.05	0	−0.012	0.009
1 0.5	−0.0005	0.0033	0.1937	0.9990	0.05	0	−0.011	0.010

### Limitations and future perspectives

The synthesis of *Punica granatum* leaf–derived CuO nanoparticles and their incorporation into calcareous AAMs was evaluated only under controlled laboratory conditions. Scale-up issues such as raw-material variability, batch reproducibility, and industrial processing routes were not tested. The study also focused on compressive strength and microstructural observations. Other critical durability parameters, such as freeze and thaw resistance, chloride and sulfate attack, and carbonation, were not assessed in the analysis. The composites produced were studied with cold pressing, whereas the long-term stability, dimensional changes, and phase changes were not assessed. Only one type of calcareous precursor and one nanoparticle loading strategy were used for this study. The results obtained cannot be generally explained for other waste-derived carbonates or different oxide nanoparticles synthesized. The process of green synthesis reduces chemical hazards, potential leaching of the Cu species; however, the life cycle assessment (LCA) analysis is a major limitation that will be performed for the future perspective of the study for the process of mass intensity, or green chemistry metrics, to rigorously validate the environmental advantages of the green approach employed for this study. Parameters such as energy consumption, waste generation, or comparative toxicity will be analyzed to substantiate the sustainability benefits of the produced materials. The long-term suspension stability in the activator was not assessed, which is a limitation for the study, and in the future, by preparing the suspension before the mixing time.

### Future perspectives

The evaluation of the resistance of the synthesized materials to aggressive environments, thermal cycling, and life cycle in order to confirm long-term performance will be performed. Other systematic variations method for analysis of CuO content, particle size distribution, and mixing routes to maximize mechanical and durability gains while minimizing cost will be employed. Other abundant botanical extracts and co-doping with hybrid nanomaterials for multifunctional properties will be used to observe their advantages for the formed matrix. Quantification of the environmental impacts, carbon savings, and potential ecotoxicity of CuO release will be evaluated and validated for the sustainability claims of the green synthesis approach. The transition from lab-scale cold-pressed samples to pilot-scale blocks, panels, or pavers, including structural testing and real-environment exposure trials, will be conducted to validate the environmental performance of the materials. Investigation of antimicrobial, photocatalytic, or thermal conductivity benefits of CuO nanoparticles in AAMs, which could open pathways to multifunctional construction materials, will be analyzed. Parameters such as energy consumption, waste generation, or comparative toxicity will be analyzed to substantiate the sustainability benefits of the produced materials.

## Conclusion

This study synthesized CuO nanoparticles using pomegranate (*Punica granatum*) leaf extract via a green synthesis approach. The resulting CuO nanoparticles were then incorporated into alkali-activated calcareous-based materials to evaluate their effects on compressive strength, porosity, water absorption, and microstructure. This study is beneficial in ensuring a sustainable approach is carried out where the use of toxic chemicals is reduced, and the green synthesized nanoparticles enhance the performance of the AAMs, making them highly sustainable. The key findings suggested that CuO nanoparticles with an average size of approximately 78 nm were successfully synthesized using the green synthesis. A zeta potential of −21 mV was recorded for the nanoparticles after sintering, indicating sufficient electrostatic repulsion to prevent significant agglomeration. Minor impurities were detected in the CuO nanoparticles, as evidenced by UV-Vis spectroscopy, XRD, and FTIR analyses. The sintering process effectively oxidized the copper particles, resulting in a highly crystalline CuO structure. TEM revealed that the CuO nanoparticles had a semi-spherical morphology with particle sizes below 100 nm. The incorporation of 0.25% and 0.5% CuO nanoparticles into the AAM enhanced the compressive strength at both one and seven days compared to the control mix. However, the mix containing 1% CuO nanoparticles exhibited a lower strength on day one but surpassed the control in strength by day seven. Adding 0.5% CuO nanoparticles based on compressive strength was optimum, which increased the compressive strength by 37.3% (p value <0.0001) on the 7th day compared to the control mix. The incorporation of CuO nanoparticles reduced the porosity and water absorption of the AAMs; nevertheless, statistical analysis at 7 days revealed no significant changes. The combined results from XRD, FTIR, SEM, and EDS analyses indicate that the CuO nanoparticles did not hinder the alkali activation process. Instead, they reinforced the matrix and enhanced the material’s overall properties. Excessive crystals were seen at higher doses of the CuO nanoparticles in the AAMs. The use of the plant extract as a natural alternative to harsh chemicals, typically in nanoparticle synthesis, and incorporating it with the AAMs makes the material environmentally friendly and sustainable.

**Research Highlights**

- The green synthesized *Punica granatum* leaf extract was used to synthesize semi-spherical CuO nanoparticles having nano sizes less than (<100 nm) to form CuO nanoparticles with a zeta potential of −21 mV, ensuring colloidal stability.
- CuO nanoparticles were incorporated into calcareous AAMs via cold-pressing, which led to an increase in compressive strength of about 37.3% at 7 days with the optimal dosage of 0.5 wt. %.
- There was a decrease in porosity and water absorption across all CuO nanoparticle content; however, the ANOVA did not show a significant result at the 7^th^ day. The result also suggested that nanoparticle-induced densification of the matrix.
- The combination of green-synthesized CuO nanoparticles with cold-pressed calcareous AAMs provides a sustainable, low-energy route to enhance the mechanical and durability properties of matrices.

This study represents the first report on green-synthesized CuO nanoparticles from *Punica granatum* leaf extract for reinforcement in calcareous AAMs, highlighting a novel pathway for eco-efficient construction materials.
